# Can risk assessment predict suicide in secondary mental healthcare? Findings from the South London and Maudsley NHS Foundation Trust Biomedical Research Centre (SLaM BRC) Case Register

**DOI:** 10.1007/s00127-018-1536-8

**Published:** 2018-06-02

**Authors:** Javier-David Lopez-Morinigo, Andrea C. Fernandes, Hitesh Shetty, Rosa Ayesa-Arriola, Ashraful Bari, Robert Stewart, Rina Dutta

**Affiliations:** 1 Department of Psychosis Studies, Institute of Psychiatry, Psychology and Neuroscience, King’s College London, De Crespigny Park, PO Box 68, London, SE5 8AF UK; 2 CAS Behavioural Health, London, UK; 3 Department of Psychological Medicine, Institute of Psychiatry, Psychology and Neuroscience, King’s College London, London, UK; 4 South London and Maudsley NHS Foundation Trust, London, UK; 5 Department of Psychiatry, Marqués de Valdecilla University Hospital, IFIMAV, School of Medicine, University of Cantabria, Santander, Spain; 6 Centro Investigación Biomédica en Red de Salud Mental (CIBERSAM), Madrid, Spain

**Keywords:** Suicide, Risk assessment, Secondary mental healthcare, Mental pain

## Abstract

**Purpose:**

The predictive value of suicide risk assessment in secondary mental healthcare remains unclear. This study aimed to investigate the extent to which clinical risk assessment ratings can predict suicide among people receiving secondary mental healthcare.

**Methods:**

Retrospective inception cohort study (*n* = 13,758) from the South London and Maudsley NHS Foundation Trust (SLaM) (London, UK) linked with national mortality data (*n* = 81 suicides). Cox regression models assessed survival from the last suicide risk assessment and ROC curves evaluated the performance of risk assessment total scores.

**Results:**

Hopelessness (RR = 2.24, 95% CI 1.05–4.80, *p* = 0.037) and having a significant loss (RR = 1.91, 95% CI 1.03–3.55, *p* = 0.041) were significantly associated with suicide in the multivariable Cox regression models. However, screening statistics for the best cut-off point (4–5) of the risk assessment total score were: sensitivity 0.65 (95% CI 0.54–0.76), specificity 0.62 (95% CI 0.62–0.63), positive predictive value 0.01 (95% CI 0.01–0.01) and negative predictive value 0.99 (95% CI 0.99–1.00).

**Conclusions:**

Although suicide was linked with hopelessness and having a significant loss, risk assessment performed poorly to predict such an uncommon outcome in a large case register of patients receiving secondary mental healthcare.

**Electronic supplementary material:**

The online version of this article (10.1007/s00127-018-1536-8) contains supplementary material, which is available to authorized users.

## Introduction

Every year, almost one million people die from suicide across the world [1], which appears to have increased since the start of the 2007 economic recession [2]. Indeed, suicide represents one of the three leading causes of death in the most economically productive age group (15–44 years) [3]. Of concern, suicide rates in the UK have shown no reduction over the past 5 years [4].

Up to 90% of people who complete suicide are found to have had a ‘psychiatric disorder’ [5], contributing to 47–74% of the population risk of suicide, with half of people completing suicide meeting retrospectively applied criteria for depression [6, 7]. Although it could be envisaged that secondary mental health services may play a crucial role in ‘suicide prevention’ [8], over two-thirds of those who take their lives in the UK have not received secondary mental healthcare in the year before death [4].

Risk assessment in mental health services might conceivably help reduce suicide rates, and the UK Department of Health [9] and 2004 NICE guidelines [10] recommended the use of structured clinical risk assessments. However, completed suicide thankfully remains a very uncommon event, and two early studies warned of the high number of false positives picked up to detect the majority of suicides using this approach [11, 12]. In particular, even a hypothetical test with a sensitivity and specificity of 99% in a high-risk population (defined as a suicide rate of 250/100,000/years) cannot predict suicide beyond a 20% level of efficiency [11]. Consistent with this, recent meta-analyses have concluded that risk scales have a limited role in predicting suicidal behaviour [13–15], although there are important issues of between-study heterogeneity [13]. In keeping with these meta-analyses, a 2017 multicentre study in the UK [16] replicated the limited use of risk scales to predict repeated self-harm, which is in line with our previous report on risk assessment and suicide by patients with schizophrenia spectrum disorders under secondary mental healthcare [17]. However, using risk assessment scales continues to be common clinical practice [18].

In addition, the extent to which risk assessment can predict suicide mortality (rather than ‘self-harm’, ‘suicide attempts’ or ‘suicidal behaviour’) in a large sample of mental health service users irrespective of diagnosis, which also changes over time [19], has not been examined to date. Within this context, we investigated the performance of all full suicide risk assessments from the South London and Maudsley (SLaM) Biomedical Research Centre (BRC) Case Register (in South-East London, UK) over 2007–2015 to predict suicide. Whilst anticipating that some risk factors would be statistically associated with a higher risk of suicide, namely previous suicide attempts, suicidal ideation, hopelessness, alcohol/drugs and impulsivity, we sought to clarify positive predictive values at different levels of raised risk, as well as the extent to which a risk assessment might allow clinicians to rule out risk: i.e., the extent to which ‘low-risk’ patients would not end their lives.

## Methods

### Participants

As stated, the sample was derived from the SLaM BRC Case Register. SLaM is one of Europe’s largest mental health services, providing secondary mental healthcare to four boroughs in South-East London (UK): Lambeth, Southwark, Lewisham and Croydon. Approximately, 1.23 million inhabitants reside in this geographic catchment area, which as a whole was found to be comparable with other populations of London in terms of age, gender, education and socio-economic status distributions [20, 21]. Fully electronic health records have been in use across all SLaM services since 2006, and in 2007–2008, the Clinical Record Interactive Search (CRIS) system was built which renders de-identified copies of records available for research use with appropriate governance structures [20]. CRIS received ethical approval as an anonymised data resource for secondary analyses from the Oxford C Research Ethics Committee (reference: 08/H0606/71+5), and currently accesses data on over 300,000 patients [21]. The same research ethics approval also covers the pseudonymised linkage between CRIS data and those from the Office for National Statistics (ONS) in April 2015 [22], which registers all deaths in the UK and the official cause of death, including suicide and the method of suicide according to ICD-10 classification [23].

Those patients who had received SLaM care (i.e., had at least one face-to-face contact with a clinical member of staff) over the period from 1st January 2007 to 1st April 2015 and had at least one suicide risk assessment documented within the study period were included. Those who died from suicide within the study period were compared with those who did not. The analysis described here was based on a surveillance period from 1 January 2007 to 1 April 2015, the rationale being that the electronic clinical records coverage became complete across all SLaM services during 2006 and, at the time of the analysis, the last death certification linkage had been accomplished in the beginning of April 2015.

### Measures

#### Risk assessment

‘Full risk assessment’ is a compulsory target across the Trust when ‘high risk’ is determined from a ‘brief risk assessment’, which is mandatory for all active cases. All patients who have been seen by a member of clinical staff have a ‘brief risk assessment’ documented, which is a narrative record of the patient’s risk: (1) to one’s self; (2) to others and (3) from others. If the patient is deemed at ‘high’ risk in any of these domains, a ‘full risk assessment’ needs to be completed and updated over time, which consists of a structured assessment taking the form of present/absent tick-boxes enquiring about widely recognised risk factors for three major clusters: suicide, violence and self-neglect. Full risk assessment is entered into CRIS as structured information separately from clinical free-text entries. Hence, information on those who had a ‘full risk assessment’ documented (compared to those without ‘full risk assessment’, including those with ‘partial’ risk assessment, i.e., only some items out of the 15-item full suicide risk assessment) can be reliably extracted from CRIS. For the purposes of this study, only those patients with a ‘full suicide risk assessment’ were included, that is, those patients with ratings on the 15 items included in the full suicide risk assessment, which is available in Appendix 1 (supplementary material available online). Positive responses can be summed to create total scores, i.e., the higher the score the greater the suicide risk, which yielded good internal consistency (Cronbach α coefficients of 0.69) [24]. The most recent full suicide risk assessment was considered for this study.

#### Demographic and clinical covariates

Demographic and clinical covariates included age at the time of risk assessment, gender, ethnicity, religion, marital status, employment status, social deprivation and ICD-10 diagnosis [23].

Social deprivation was scored through an anonymous link created in CRIS between lower super output area residence code of the latest permanent address (a geographic unit comprising approximately 400 households) and summary data for that area from 2007 UK Census output. Thus, the Index of Multiple Deprivation (IMD) is derived from seven domains: income, employment, health, education, housing and services, crime and environment [25].

ICD-10 diagnoses [23] were reached by consensus by the treating multidisciplinary team, including input from a senior consultant psychiatrist. Specifically, several clinically meaningful categories were created as follows: ‘organic mental disorders’ (F0), ‘substance abuse’ (F1), ‘schizophrenia spectrum disorders’ (F2), ‘mood disorders’ (F3), ‘neurotic disorders’ (F4) and ‘all others’ (F5–F7).

#### Suicide method

Suicide method was ascertained using death certificate [22] ICD-10 codes [23] and the following groups were used to define this: poisoning—X64; hanging—X70; drowning—X71; cutting—X78; jumping (either from high place or in front of a vehicle)—X80, X81; suicide by unspecified means—X84; and undetermined cause of death—Y10-34. Those with an ‘undetermined cause of death’ code were considered as suicides, because in the UK most ‘open verdicts’ have been reported as very likely to be suicides [26].

### Statistical analysis

First, for descriptive purposes, for all SLaM ‘active’ service users (i.e., at least one face-to-face contact with a staff member) over the study period (2007–2015), we investigated suicide rates differences between those with/without full risk assessment.

Second, in those with at least one full risk assessment documented (i.e., the study sample), risk assessment individual items and total scores, as independent variables, entered into Kaplan–Meier survival analyses and Cox regression models [27], respectively, to investigate associations with time to suicide. Proportional hazards assumptions were checked as standard for Cox procedures and no evidence of violation was found, i.e., the survival curves for two strata (determined by the values for the covariates) had ‘hazard’ functions, which were ‘proportional’ (or constant) over time. Age, gender, religion, employment and marital status, ethnic group, IMD and primary psychiatric diagnosis were entered as covariates. For the survival analyses, the follow-up period began at the time of the last risk assessment and the end date was the date of death (including suicide) or the censoring point (last face-to-face contact, date of death from non-suicide causes or 1st April 2015, whichever came sooner).

In addition, receiver operating characteristic (ROC) curves [28], which compare the true positive rate (i.e., sensitivity) with the false positive rate (i.e., ‘1-specificity’) at different cut-off points for the parameter (risk assessment total score in our study), were plotted to analyse the performance of risk assessment total scores to predict suicide. In particular, sensitivity, specificity, positive and negative predictive values, the area under the curve (AUC), likelihood ratios (positive and negative) and diagnostic odds ratio (OR) were investigated for the best cut-off point, including 95% confidence intervals (CI) for each statistic at each risk assessment total score. Positive and negative predictive values are the probability that subjects with a positive (high risk) result will truly have the outcome of interest (in this study, death from suicide), and the probability that subjects with a negative (low risk) result will not have such an outcome, respectively. The AUC is a measure of how well risk assessment total score can distinguish those who will die from suicide from those who will not. Likelihood ratios are the likelihood that a given test result would be expected in a patient who took his/her life compared to the likelihood that same result would be expected in a patient who did not end his/her life. Diagnostic ORs are the ratio of the odds of the test being positive (high risk) if the subject ended his/her life relative to the odds of the test being positive if the subject did not die from suicide.

A significant level of 5% (two-tailed) was set for all the above analyses, which were performed using the statistical package R (version 3.20) [29].

## Results

### Study sample

Over 2007–2015, there were 99,507 SLaM ‘active’ cases, i.e., those who had at least one face-to-face contact with a SLaM staff member over that period (2007–2015), of whom 358 were ascertained as having died by suicide. Of all these active SLaM service users, 13,758 subjects had all suicide-related items completed on a full risk assessment, and a further 1409 had incomplete data (with at least one item rated). Of the 13,758 individuals, who formed the study sample, 81 were recorded as having died by suicide. Taking into account the observation time of each patient, the analysed sample contributed to 80,769.17 person-years, which yielded a suicide rate of 100.28/100,000 person-years. In those with a partial risk assessment (*n* = 1409), the suicide rate was 51.60/100,000 person-years, while for those with no risk assessment completed (*n* = 80,340), the suicide rate was 88.34/100,000 person-years. These differences were not statistically significant (*X*^2^ = 6, *df* = 4, *p* = 0.19).

The baseline demographic and clinical characteristics of the sample (*n* = 13,758) and differences between those who took their lives and those who did not are presented in Table 1. Although there was a higher male predominance in the suicide completers group than in those who did not end their lives (OR = 1.67, 95% CI 1.04–2.69, *p* = 0.03), no further significant differences emerged in age at first presentation, religion, marital status, ethnicity, living status, employment, social deprivation, first language (English vs. all others) and ICD-10 diagnoses. Hanging was the most common suicide method (*n* = 28). Twenty-one subjects received an undetermined cause of death. There were no suicides by firearms.

**Table 1:** Demographics and clinical characteristics of the sample

	Suicides *N* = 81	Non-suicides *N* = 13,678		*p* value
	Mean ± SD	Mean ± SD		
Age at risk assessment (years)	41.3 ± 12.2	40.6 ± 11.5		0.60
Social deprivation	28.6 ± 13.4	28.6 ± 12.3		0.98
	*n* (%)	*n* (%)	OR (95% CI)	
Gender (males)	56 (69.1)	7823 (57.2)	1.67 (1.04–2.69)	0.03
Marital status (unmarried)	72 (88.9)	11,909 (87.1)	1.10 (0.55–2.21)	0.78
Employment status (unemployed)	30 (37.0)	4662 (34.0)	1.57 (0.48–5.14)	0.46
Living status (alone)	19 (23.5)	3093 (22.6)	1.04 (0.57–1.89)	0.90
Religion (yes)	20 (24.7)	3177 (23.2)	1.00 (0.60–1.67)	0.98
Ethnicity				
White	50 (61.7)	6916 (50.6)	1.85 (1.02–3.35)	0.04
Black	14 (17.3)	3525 (25.8)	Ref.	
Others	17 (21.0)	3237 (23.7)	1.37 (0.67–2.78)	0.38
First language (non-English)	40 (49.4)	5971 (43.7)	1.53 (0.99–2.37)	0.06
Diagnosis				
Organic disorders	1 (23.0)	270 (2.0)	Ref.	
Substance use disorders	10 (12.3)	1232 (9.00)	1.95 (0.25–15.2)	0.53
Schizophrenia spectrum	31 (38.3)	5713 (41.8)	1.28 (0.17–9.34)	0.81
Mood disorders	24 (26.6)	2643 (19.3)	2.28 (0.30–16.9)	0.42
Neurotic disorders	5 (6.17)	860 (6.30)	1.60 (0.19–13.7)	0.67
Other diagnoses	10 (12.3)	2960 (21.6)	0.85 (0.10–6.60)	0.87
Method				
Hanging	28 (34.6)			
Intoxication	6 (7.4)			
Jumping	3 (3.7)			
Unspecified means	16 (19.7)			
NA	7 (8.6)			
Undetermined cause of death	21 (25.9)			

### Risk assessment factors

Differences in individual risk assessment items between suicide completers and non-completers (Kaplan–Meier survival analyses) are presented in Table 2. The following items were significantly associated with an increased risk of suicide: previous suicide attempts (RR = 2.46, 95% CI 1.51–4.01, *p* < 0.001), previous use of a violent method (RR = 2.46, 95% CI 1.53–3.97, *p* < 0.001), plans to end life (RR = 3.37, 95% CI 1.98–5.77, *p* < 0.001), suicidal ideation (RR = 2.06, 95% CI 1.30–3.31, *p* = 0.002), hopelessness (RR = 2.79, 95% CI 1.78–4.37, *p* < 0.001), distress (RR = 1.66, 95% CI 1.07–2.60, *p* = 0.024), lack of control over life (RR = 2.13, 95% CI 1.36–3.35, *p* < 0.001), impulsivity (RR = 1.64 95% CI 1.05–2.57, *p* = 0.030), having a significant loss (RR = 1.88, 95% CI 1.20–2.96, *p* = 0.006), disengagement (RR = 1.85, 95% CI 1.17–2.91, *p* = 0.007) and recent hospital discharge (RR = 1.93, 95% CI 1.20–3.14, *p* = 0.007).

**Table 2:** Unadjusted univariate analyses: risk assessment items

Risk factor	*N*	Events expected	Events observed	Log-rank test	RR (95% CI)	*p* value
Previous suicide attempts						
Absent	7004	40	24	14.1	2.46 (1.51–4.01)	< 0.001
Present	5657	33	49			
Violent method						
Absent	9167	50.7	37	14.7	2.46 (1.53–3.97)	< 0.001
Present	3069	17.3	31			
Plan to end life						
Absent	11,551	64.5	53	22.5	3.37 (1.98–5.77)	< 0.001
Present	1258	6.5	18			
Suicidal ideation						
Absent	10,583	61.7	51	9.4	2.06 (1.30–3.31)	0.002
Present	2843	15.3	26			
Hopelessness						
Absent	9419	55.1	37	21.7	2.79 (1.78–4.37)	< 0.001
Present	3811	20.9	39			
Distress						
Absent	8617	51.5	42	5.1	1.66 (1.07–2.60)	0.024
Present	4651	26.5	36			
No control over life						
Absent	9290	55.1	42	11.3	2.13 (1.36–3.35)	< 0.001
Present	3576	20.9	34			
Alcohol/drugs						
Absent	7834	44.1	36	3.8	1.60 (0.99–2.51)	0.051
Present	4971	27.9	36			
Impulsivity						
Absent	7292	43.5	34	4.7	1.64 (1.05–2.57)	0.030
Present	5636	33.5	43			
Living alone						
Absent	8358	48.3	46	0.3	1.12 (0.72–1.76)	0.603
Present	4949	30.7	33			
Poor physical health						
Absent	9616	56.2	56	0	1.01 (0.61–1.67)	0.964
Present	3431	20.8	21			
Significant loss						
Absent	8243	50	39	7.5	1.88 (1.20–2.96)	0.006
Present	4256	24	35			
Disengagement						
Absent	9841	58.3	48	7.2	1.85 (1.17–2.91)	0.007
Present	3277	19.7	30			
Recent hospital discharge						
Absent	10,973	65.1	56	7.3	1.93 (1.20–3.14)	0.007
Present	2399	13.9	23			
Family history						
Absent	5921	32.88	35	1.2	0.46 (0.11–1.90)	0.268
Present	730	4.12	2			

Cox regression analyses of the relationship between those risk assessment factors significantly associated with risk of suicide (see Table 2) are presented in Table 3. The left column shows the results only after adjusting the analyses for gender, which had been the only significant baseline characteristic associated with suicide risk (see Table 1). All the risk factors remained significant (Table 3, left column). The right column of Table 3 presents coefficients from a model containing all factors; in this, only hopelessness (RR = 2.24, 95% CI 1.05–4.80, *p* = 0.037) and having a significant loss (RR = 1.91, 95% CI 1.03–3.55, *p* = 0.041) remained statistically significant predictors of suicide. All the other associations, apart from that with previous suicide attempts (which showed a strengthened coefficient but wider confidence intervals), were substantially attenuated.

**Table 3:** Adjusted Cox regression analyses: risk assessment items

Individual items	RR^a^ (95% CI)	*p* value	RR^b^ (95% CI)	*p* value
Gender (male)	1.67 (1.04–2.67)	0.03	1.70 (0.95–3.02)	0.077
Suicidal history	**1.67 (1.04–2.67)**	< **0.001**	2.00 (0.89–4.53)	0.094
Violent method	**2.58 (1.58–4.21)**	< **0.001**	1.31 (0.65–2.65)	0.453
Plan to end life	**2.47 (1.53–3.98)**	< **0.001**	1.20 (0.54–2.63)	0.657
Suicidal ideation	**2.13 (1.32–3.41)**	**0.002**	0.82 (0.40–1.67)	0.577
Hopelessness	**2.90 (0.85–4.55)**	< **0.001**	**2.24 (1.05–4.80)**	**0.037**
Distress	**1.72 (1.10–2.70)**	**0.020**	0.90 (0.48–1.68)	0.746
No control over life	**2.21 (1.40–3.48)**	< **0.001**	0.95 (0.47–1.91)	0.881
Impulsivity	**1.65 (1.05–2.58)**	**0.029**	1.05 (0.56–1.97)	0.875
Significant loss	**1.95 (1.23–3.07)**	**0.004**	**1.91 (1.03–3.55)**	**0.041**
Disengagement	**1.85 (1.17–2.92)**	**0.008**	1.16 (0.61–2.20)	0.653
Recent hospital discharge	**1.97 (1.21–3.21)**	**0.006**	1.45 (0.77–2.74)	0.247

Bold values indicate statistically significance *p* < 0.05

^a^Analysis adjusted for gender only

^b^Fully adjusted analysis

### Risk assessment overall performance

**Fig. 1:**
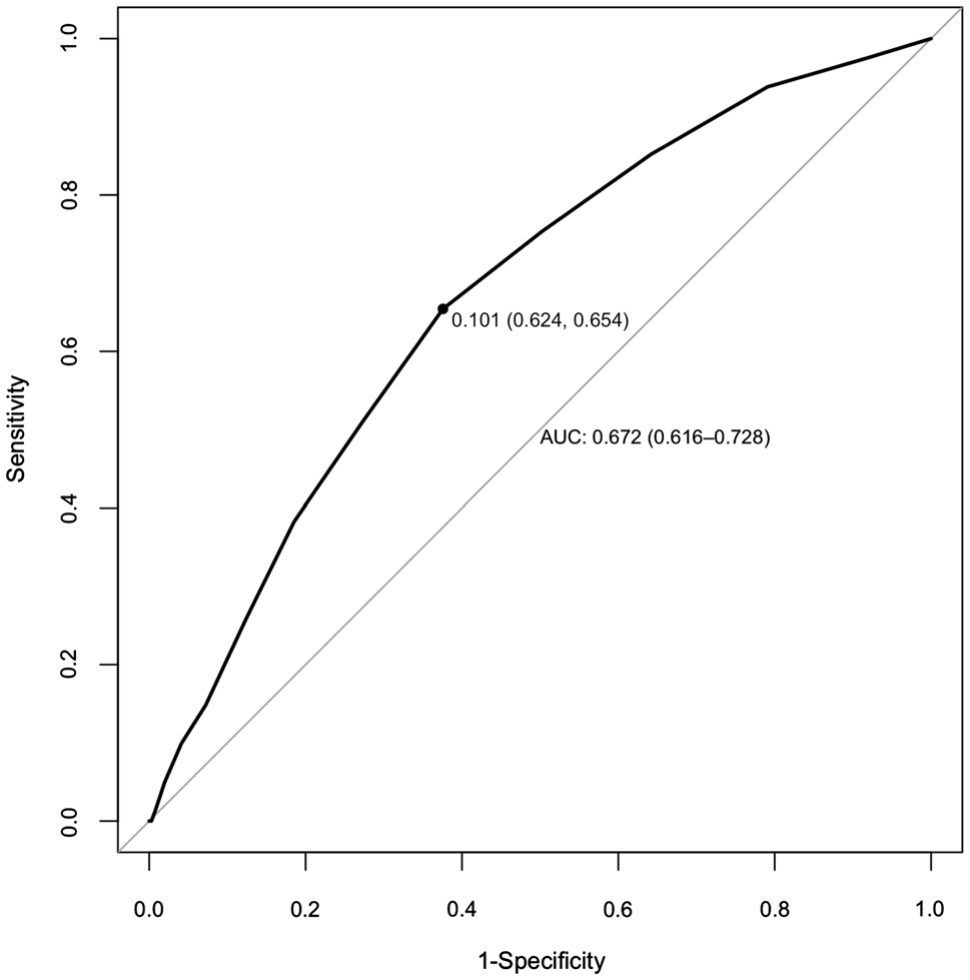
ROC curve for risk assessment total scores

The diagnostic accuracy statistics (and 95% CI) for each risk assessment total score are detailed in Table 4. ROC curve analyses for risk assessment total scores, which are shown in Fig. 1, found the optimal cut-off point to be 4–5 (above which the risk would be ‘high’; below which the risk would be ‘low’), with a sensitivity of 0.65 (95% CI 0.65–0.76), specificity of 0.62 (95% CI 0.62–0.63) and an AUC of 0.67 (95% CI 0.62–0.73). The positive and negative predictive values were 0.01 (95% CI 0.01–0.01) and 0.99 (95% CI 0.99–1.00), respectively.

**Table 4:** Risk assessment total scores (number of risk factors present) and diagnostic accuracy statistics with 95% confidence intervals

Risk assessment (Total score)	Suicides	Non-suicides	Sensitivity, % (95% CI)	Specificity, % (95% CI)	Positive predictive value, % (95% CI)	Negative predictive value, % (95% CI)	Likelihood ratio, + (95% CI)	Likelihood ratio, − (95% CI)	Diagnostic OR (95% CI)
0	2	1113	98 (91–100)	8 (8–9)	1 (0–1)	100 (99–100)	1.1 (1.0–1.1)	0.3 (0.1–1.2)	3.5 (0.9–14.3)
1	3	1748	94 (86–98)	21 (20–22)	1 (1–1)	100 (100–100)	1.2 (1.1–1.3)	0.3 (0.1–0.7)	4.0 (1.6–9.9)
2	7	2042	85 (76–92)	36 (35–37)	1 (1–1)	100 (100–100)	1.3 (1.2–1.5)	0.4 (0.2–0.7)	3.2 (1.7–5.9)
3	8	1909	75 (64–84)	50 (49–51)	1 (1–1)	100 (100–100)	1.5 (1.3–1.7)	0.5 (0.3–0.7)	3.02 (1.8–5.0)
4	8	1727	65 (54–76)	62 (62–63)	1 (1–1)	100 (99–100)	1.7 (1.5–2.0)	0.5 (0.4–0.7)	3.1 (2.0–5.0)
5	12	1443	51 (39–62)	73 (72–74)	1 (1–1)	100 (99–100)	1.9 (1.5–2.3)	0.7 (0.5–0.8)	2.8 (1.8–4.3)
6	10	1161	38 (28–50)	81 (81–82)	1 (1–2)	100 (99–100)	2.1 (1.6–2.7)	0.8 (0.6–0.9)	2.7 (1.73–4.3)
7	10	836	26 (17–37)	88 (87–88)	1 (1–2)	100 (99–100)	2.1 (1.4–3.0)	0.8 (0.7–1.0)	2.5 (1.5–4.1)
8	9	712	15 (8–24)	93 (92–93)	1 (1–2)	100 (99–100)	2.0 (1.2–3.5)	0.9 (0.8–1.0)	2.23 (1.2–4.1)
9	4	427	10 (4–19)	96 (96–96)	1 (1–3)	100 (99–100)	2.4 (1.2–4.7)	0.9 (0.8–1.0)	2.6 (1.2–5.3)
10	4	291	5 (1–12)	98 (98–98)	1 (1–4)	100 (99–100)	2.5 (0.9–6.6)	1.0 (0.9–1.0)	2.6 (0.9–7.1)
11	3	167	1 (0–7)	99 (99–99)	1 (0–5)	100 (99–100)	1.7 (0.2–11.7)	1.0 (1.0–1.0)	1.6 (0.2–12.1)
12	1	64	0 (0–4)	1 (1–1)	0 (0–9)	99 (99–100)	0 (0–0)	1.0 (1.0–1.0)	NA
13	0	28	NA	NA	NA	NA	NA	NA	NA
14	0	10	NA	NA	NA	NA	NA	NA	NA

## Discussion

### Main findings

We drew data from a large case register of patients receiving secondary mental healthcare in a defined catchment area over a prolonged period (2007–2015) linked with national mortality data and we tested the extent to which structured risk assessment items (individual risk factors and overall scores) predicted suicide. First, as expected, we identified a high number of suicides in a population of patients in secondary mental healthcare (approximately 100.28/100,000 person-year), approximately tenfold higher than in the general population (10.9/100,000 person-year) [22]. Second, we found that although a number of risk factors were significantly associated with suicide in the bivariate analyses (namely, being male, previous suicide attempts, previous use of violent methods, plans to end life, suicidal ideation, distress, lack of control over life, impulsivity, disengagement from services/non-compliance with medication and recent hospital discharge), only hopelessness and having a significant loss remained independent and statistically significant predictors of suicide in the multivariable regression models. Third, overall risk assessment performed poorly to predict suicide in a large sample of mental health service users, which was in line with our expectations and recent literature [13–15].

### Comparison with previous literature

Of relevance, we did not find a protective effect of completing suicide risk assessment on reducing suicide rates as previously suggested [10], which was in line with further reviews of the NICE guidelines [30]. It might seem intuitive to speculate that those patients with a risk assessment documented are deemed at a higher risk of suicide by the treating team and they are more likely to receive a higher level of input and caution in management. It remains unanswerable what may have happened if these ‘high risk’ individuals had not been administered a risk assessment and to research this would raise ethical issues. However, recording of risk assessment is, to some degree, circular and more likely in those patients who will be followed-up by mental health services [31]. In addition, risk assessment in this cohort may have been completed due to concerns raised regarding other clusters of risk, such as violence to others and/or self-neglect [24]. Moreover, risk assessment completion rates may have been affected by the patient’s legal status in some cases, which was not evaluated in this study. For instance, those receiving care under restriction, hence subject to the UK Mental Health Act 1983 (amended 2007) [32] may be more likely to have a risk assessment documented. In this regard, our findings are of major relevance from a human rights perspective, since these individuals may have been ‘forced’ to undertake an assessment which appears to have a limited value, which would also go against the 2015 UK Code of Practice [33].

In terms of risk factors, we replicated the association of being male [1, 34], previous suicide attempts [35], previous use of violent methods [5], plans to end life and suicidal ideation [36], hopelessness [37, 38], distress, lack of control over life and impulsivity [39], having a significant loss [40], disengagement from services/non-compliance with medication and recent hospital discharge [4] with suicide. Consistent with previous models of suicide [41, 42], only hopelessness and having a significant loss remained significant. However, it should be noted that childhood trauma, which was not evaluated by our risk assessment questionnaire, was found to have greater effects on suicidality than depression and related variables [43], hence it should become part of routine clinical suicide risk assessment.

Over four decades ago, hopelessness was defined as ‘the cognitive element of negative expectations’ [37], which was also demonstrated to be the strongest predictor of suicide in outpatients [38], and this we replicated in our results. Hence, our findings agree, in part, with Mann’s diathesis-stress model of suicide [42] regarding the role of hopelessness, although impulsivity [39] was not significantly associated with suicide in our cohort. It should be noted, however, that impulsivity, as measured by the SLaM risk assessment, might refer to a different construct. On the other hand, we did find that having a significant loss was a predictor of suicide independently of other factors, which, in addition to the role of hopelessness, was consistent with the classic theory of ‘suicide as psychache’ [41]. Of note, the conceptualization of suicide as the consequence of ‘mental pain or psychache’ has been recently reconsidered [44] in light of decades of relatively unsuccessful neurobiological research [45] based on Mann’s model of suicide [42]. Indeed, over 90% of suicide attempters report ‘mental pain’ [46], which is frequently the consequence of a bereavement [40], which is of particular concern after surviving the suicide of a love one [47]. Moreover, ‘mental pain’ appears to be a contributor to suicide independently of depression [48], which is in full agreement with our results. Indeed, the relationship between suicide and ‘mental illness’ (from a psychiatric perspective) may be weaker than previously thought, especially in Western countries [44]. In keeping with this, neither medication compliance nor (psychiatric) diagnosis were associated with suicide in our large cohort of mental health service users, which may provide further support for a non-medical approach to suicide [49]. Hence, those patients receiving secondary mental healthcare at risk of suicide may particularly benefit from psychological therapies, as recommended by the UK NICE guidelines for depression [50], although not frequently offered [51].

In addition, overall risk assessment showed poor predictive validity, which was unsurprising, given the rarity of the outcome. In particular, high sensitivity was reached at the price of low specificity (i.e., a very high number of false positives to identify most of suicides) and vice versa, i.e., reducing the number of false positives (high specificity) occurred at the expenses of too many false negatives (low sensitivity), thus preventing high-risk patients from care and treating ‘healthy’ people unnecessarily, which was in full agreement with early literature [11, 12].

In keeping with this, for the most optimal cut-off point (4–5), a very low positive predictive value (1%) and very high negative predictive value (99%) emerged from the analyses. In other words, in this ‘low-risk’ group (those with less than four risk factors), there would be still 20 suicides (approximately one quarter of those who ended their lives). On the other hand, 6988 individuals (50.8% of the total sample) would be classified as ‘high-risk’ patients, although only 61 of these subjects took their lives. The question arises. Is it, therefore, worth managing so many patients as ‘suicidal’ to prevent a few deaths? More specifically, in times of financial constraints, should so many patients receive high levels of care such as unnecessary admissions to hospital?

These findings were consistent with a 2017 literature review on ‘the limitations of epistemic uncertainty’ with regard to risk assessment, whose overall poor performance appears to be due to the so-called ‘aleatory uncertainty’. In short, risk factors change ‘by chance’, which is unpredictable [18]. The concept of risk, therefore, requires a reformulation. Specifically, while suicide risk does not appear to be predictable, a more prevention-orientated approach may result in better clinical outcomes [52].

However, our findings concerning the association of hopelessness and having a significant loss with suicide, which as a whole provide some support for the ‘mental pain’ model of suicide [41], which was discussed above, still suggest that suicide may still be, to some degree, predictable.

### Future research

While we do recommend that risk assessment should remain part of our routine clinical practice, a more narrative (free-text) approach should be taken [53] to better capture aspects such as ‘mental pain’, which, based on our findings, seems to be more useful in terms of clinical risk assessment. In particular, rather than categorising patients as ‘low–medium–high’ risk, the wide range of contributing factors to risk should be detailed in relation to the individual’s mental health problems and the social context and how these factors may change dynamically over time, thus increasing or decreasing risk for a given individual, which is what matters clinically [52]. Patient information from electronic records can be easily, safely and securely de-identified to generate large datasets for secondary research [54], such as the SLaM CRIS [20, 21]. Specifically, naturalistic language processing (NLP) tools appear to be promising research instruments to extract statistically analysable clinical information from narrative electronic records, hence determining risk from free-text notes [55]. NLP techniques may assess suicide risk using information from unstructured questions with higher precision than the classic risk assessment scales [56], thus potentially capturing the presence of ‘mental pain’.

Specifically, future studies should examine whether risk assessment changes over time, particularly self-ratings shortly before suicide may increase the predictive value of risk assessment. In this regard, mobile phone and web-based text messaging may represent a useful tool to self-monitor suicide risk [57], particularly to follow-up people attempting suicide [58] and to assess risk shortly before suicidal events, including suicides. For instance, the classic suicide note may have been substituted by a message left on this new media, which clinicians should discuss with patients and carers when assessing self-harm [59]. In addition, free-text-based risk assessment, which can be researched through NLP techniques [55], may be more accurate than psychometric scales [56]. Future research should, therefore, switch the focus from long-term risk factors to short-term risk algorithms, which are more relevant to the clinician [60]. However, the evidence of the use of communication technologies in health care and public health, which is known as mobile health (mHealth) [61], on suicide prevention is limited [62].

### Strengths and limitations

The use of a large case register linked with national mortality data allowed us to investigate the role of risk assessment in predicting suicide in a large sample of patients who were receiving secondary mental healthcare within a defined geographic catchment and time period. Since most people living in South-East London requiring secondary mental healthcare receive this from NHS resources, our sample is likely to be representative. In addition, participants were followed-up for up to 9 years and, in addition to risk assessment ratings, a number of covariates were taken into account.

However, the study has some limitations to be borne in mind when drawing conclusions from the results. First, all participants were mental health service users residing in South-East London, which is an inner urban area. Hence, our findings may not generalise to people with mental health problems under primary care or those who live in rural areas. Second, the vast majority of SLaM patients (almost 90%) did not have a structured risk assessment completed and those who did may have been deemed ‘at-high-risk’ by their treating teams. In other words, it could be still speculated that assessing risk in all patients receiving care may reduce risk. Third, although only the last suicide risk assessment was considered, risk factors may have changed from the time of risk assessment to death. Also, risk assessment scores may have been affected by survival, hence those who survived for longer (and therefore received care for a more prolonged period of time) may have been rated differently. Finally, other factors such as patient’s legal status at the time of risk assessment or a history of childhood trauma, which were not evaluated in this study, may have affected both risk assessment completion rates and ratings, and the main outcome measure of this study, namely suicide.

### Implications and conclusions

Our results, therefore, support the notion that neither individual risk factors nor a combination of them, i.e., risk assessment, can adequately predict suicide in a population of patients receiving mental healthcare. Suicide is a very uncommon outcome even in a high-risk group such as patients receiving secondary mental healthcare. Our study suggests that risk assessment cannot predict suicide in the clinical setting due to its very low occurrence, which is in full agreement with recent meta-analyses [13–15], although hopelessness and having a significant loss were linked with suicide, consistent with the classic ‘mental pain’ model of suicide [41, 44, 46, 48].

Meanwhile, further research on suicide prevention [62–64] is warranted. In particular, means restriction remains the first-line strategy to prevent both high-risk groups such as patients receiving mental healthcare [17] and the general population [65] from suicide.

## Electronic supplementary material

Below is the link to the electronic supplementary material.

Supplementary material 1 (DOC 24 KB)
