# Supplementary material for: Can risk assessment predict suicide in secondary mental healthcare? Findings from the South London and Maudsley NHS Foundation Trust Biomedical Research Centre (SLaM BRC) Case Register
Source: Soc Psychiatry Psychiatr Epidemiol. 2018 Jun 2;53(11):1161–71. doi: 10.1007/s00127-018-1536-8 (PMC6208937; doi:10.1007/s00127-018-1536-8)
Supplement: Supplementary file 1 — Supplementary material 1 (DOC 24 KB) [file 127_2018_1536_MOESM1_ESM.doc]

**Appendix 1. Suicide risk assessment**

a. Does the patient have a history of suicide attempts? y/n

b. If so, did (s)he use a violent/perceived lethal method? y/n

c. Has the patient made a plan to end his/her life? y/n

d. Is the patient expressing suicidal ideation? y/n

e. Is the patient expressing feeling of hopelessness? y/n

f. Does the patient express high levels of subjective distress (from psychotic symptoms/

situations)? y/n

g. Does the patient express feelings of having no control over his/her life? y/n

h. Does the patient misuse drugs/alcohol? y/n

i. Does the patient display impulsivity? y/n

j. Does the patient live alone? y/n

k. Does the patient have poor physical health? y/n

l. Has the patient recently suffered significant loss or threat of loss? (include perceived

loss of status or role) y/n

m. Has the patient recently disengaged with care or stopped medication? y/n

n. Has the patient recently been discharged from hospital? (within the last six months) y/n

o. Does the patient have a family history of suicide? y/n
